# A Multi-Criteria Approach for Comparison of Ginger Extract and Conventional Irrigants in Root Canal Treatment

**DOI:** 10.7759/cureus.29327

**Published:** 2022-09-19

**Authors:** Hamid Reza Abdollahi-Mansoorkhani, Farzad Soleimani, Farshid Mahmoudi

**Affiliations:** 1 School of Dentistry, Endodontic Department, Islamic Azad University of Shiraz, Shiraz, IRN

**Keywords:** enterococcus faecalis, anp, naocl, ethanolic extract, antimicrobial efficacy, ginger

## Abstract

Background

Considering the importance of irrigation in dental root canal treatment, there is an urgent need to find a risk-free bioactive and antibacterial endodontic solution. *Enterococcus faecalis*, an anaerobic gram-positive coccus, has been identified as the main reason for endodontic infections. Several studies have been conducted on *E. faecalis* and periapical infection. Nowadays, plants used in traditional medicine play a role that is widely appreciated by researchers. One of these herbs is ginger which shows an acceptable antimicrobial effect on *E. faecalis*. Due to the highly crucial role that irrigation plays in the success of endodontic treatment, a comprehensive survey based on several criteria, namely, scientific, technical, and empirical, is required to address the goal of determining the best endodontic solution.

Methodology

The most important criteria are antibacterial activity, risks and hazards, cost, and availability. In this study, the analytical network process (ANP), which is a multi-criteria decision-making method, was applied to determine the best endodontic irrigant.

Results

Several alternatives were investigated using the ANP. In this study, 5.25% sodium hypochlorite (NaOCl) and 2% chlorhexidine were at the top of the list. According to the sensitivity analysis, 10% ethanolic ginger extract showed comparable results to 2.5% NaOCl.

Conclusions

To carefully prioritize endodontic irrigants a wide range of standards and criteria should be considered. Considering the low risk, great wettability, and active compounds of ginger extract, it can be a promising viable risk-free solution for root canal treatments.

## Introduction

Successful root canal treatment depends on the complete elimination of pathogenic bacteria [1]. Because antimicrobial activity plays an important role in the efficacy of endodontic irrigation used during root canal therapy, many studies have dealt with the antibacterial activity of endodontic irrigations [2,3]. *Enterococcus faecalis* has been repeatedly identified as one of the most resistant species in the oral cavity [4] and is the main microorganism associated with endodontic failure [5,6]. Herbal extracts are widely considered to be of lower risk compared with synthetic drugs; therefore, many studies have suggested them as sources of new pharmaceutical formations [7]. According to the FDA, ginger (*Zingiber officinale*, family Zingiberaceae) has been classified as Generally Recognized as Safe (GRAS). A 1 g dose of ginger powder ingested two to three times for periods up to 2.5 years did not cause any negative consequences [8]. Various studies have reported that the rhizome of ginger has strong antibacterial properties [9]. The main bioactive compounds in ginger, such as gingerols, shogaols, paradols, and terpene compounds, possess biological activities such as antibacterial, antioxidant, anti-inﬂammatory, anti-lipid, antidiabetic, analgesic, antipyretic, and anti-cancer effects [10,11]. Studies have indicated that the active components of ginger have antimicrobial activity against *Escherichia coli*, *Salmonella typhi*, and *Bacillus subtilis.* Moreover, the ethanolic extract of ginger shows the widest zone of inhibition against *S. typhi* [12,13]. Fresh ginger juice has been shown to have inhibitory action against *Aspergillus niger*, *Saccharomyces cerevisiae*, *Mycoderma *sp., and *Lactobacillus acidophilus* at 4%, 10%, 12%, and 14%, respectively [14]. Many studies have attempted to determine an effective alternative strategy for the prevention or eradication of *E. faecalis* from gaining access to the root canal system during treatment, between appointments, or even after the treatment has been completed [15,16]. Sodium hypochlorite (NaOCl) is widely used to irrigate root canals in dentistry. Despite its benefits, serious complications can result from inadvertent use due to its cytotoxic features, such as damage to the skin, oral mucosa, and eyes [17]. Therefore, there is an urgent need to explore novel bioactive compounds that are safer and biodegradable. Hence, this study aimed to analyze in vitro antibacterial activity of the ethanolic and aqueous ginger extract in comparison with NaOCl against *Enterococcus* using a multi-factorial framework. In this article, a method is presented for addressing the best endodontic solution in root canal therapy. The proposed method is the analytical network process (ANP), which is the multi-criteria decision-making method. ANP was implemented for choosing one of the irrigation between ginger extract, NaOCl, and 2% chlorhexidine (CHX). There are several techniques for gathering and integrating information and expert opinions; in this analysis, the Delphi technique was used.

## Materials and methods

To prioritize different endodontic solutions, it is important to collect and use the opinions and experiences of experts and practitioners in the related fields in addition to the international indicators and scientific criteria. The selected method for the analysis should be a subset of multi-criteria decision-making (MCDM) methods. For such an analysis, wherein there are several criteria and alternatives to achieve a goal, ANP would be the most accurate and acceptable method. Methods such as ELECTRE and TOPSIS are the conventional methods in MCDM. Because of the old techniques and the complexity or lack of access to software, they cannot defeat the accurate ANP method. The first step in the ANP method is to collect and integrate expert opinions. Various techniques are available for this purpose. Due to the possibility of dispersion and the lack of access to experts, the Delphi method was used. ANP is one of the most common MCDM methods reported by Mr. Saaty in 1970 [18]. ANP reflects natural behavior and human thought. Complex issues are examined in this method based on their interactions and are solved in a simple manner. The ANP is a qualified method that performs weight analysis with a combination of qualitative and quantitative aspects. The hidden foundation of ANP is pair-wise comparison [19]. Table 1 lists the scales that are used in the one-on-one comparison.

**Table 1:** Scales used in one-on-one comparisons in the common ANP method. ANP = analytical network process

Definition	Scale
Equally important	1
Moderately more important	3
Strongly more important	5
Very strongly more important	7
Exceedingly more important	9
Intermediate preferences	2, 4, 6, 8

A pair-wise comparison is shown in Table 2. Numbers of one-on-one comparison matrices demonstrate the priority of one criterion over another.

**Table 2:** A sample comparison table based on the proposed criteria.

	CRI.1	CRI.2	CRI.3	CRI.4
CRI.1	1	C_12_	C_13_	C_14_
CRI.2	C_21_	1	C_23_	C_24_
CRI.3	C_31_	C_32_	1	C_34_
CRI.4	C_41_	C_42_	C_43_	1

**Figure 1:**
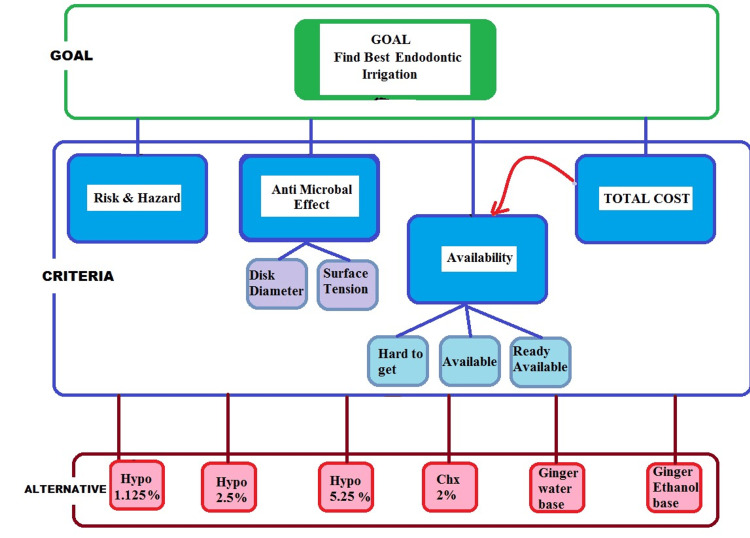
Hierarchy structure of the studied problem.

The problem is broken into a hierarchy of elements. In this way, the goal/target is placed at the top, while the main attributes are placed on the lower levels. The notes are outlined in Figure 1.

**Figure 2:**
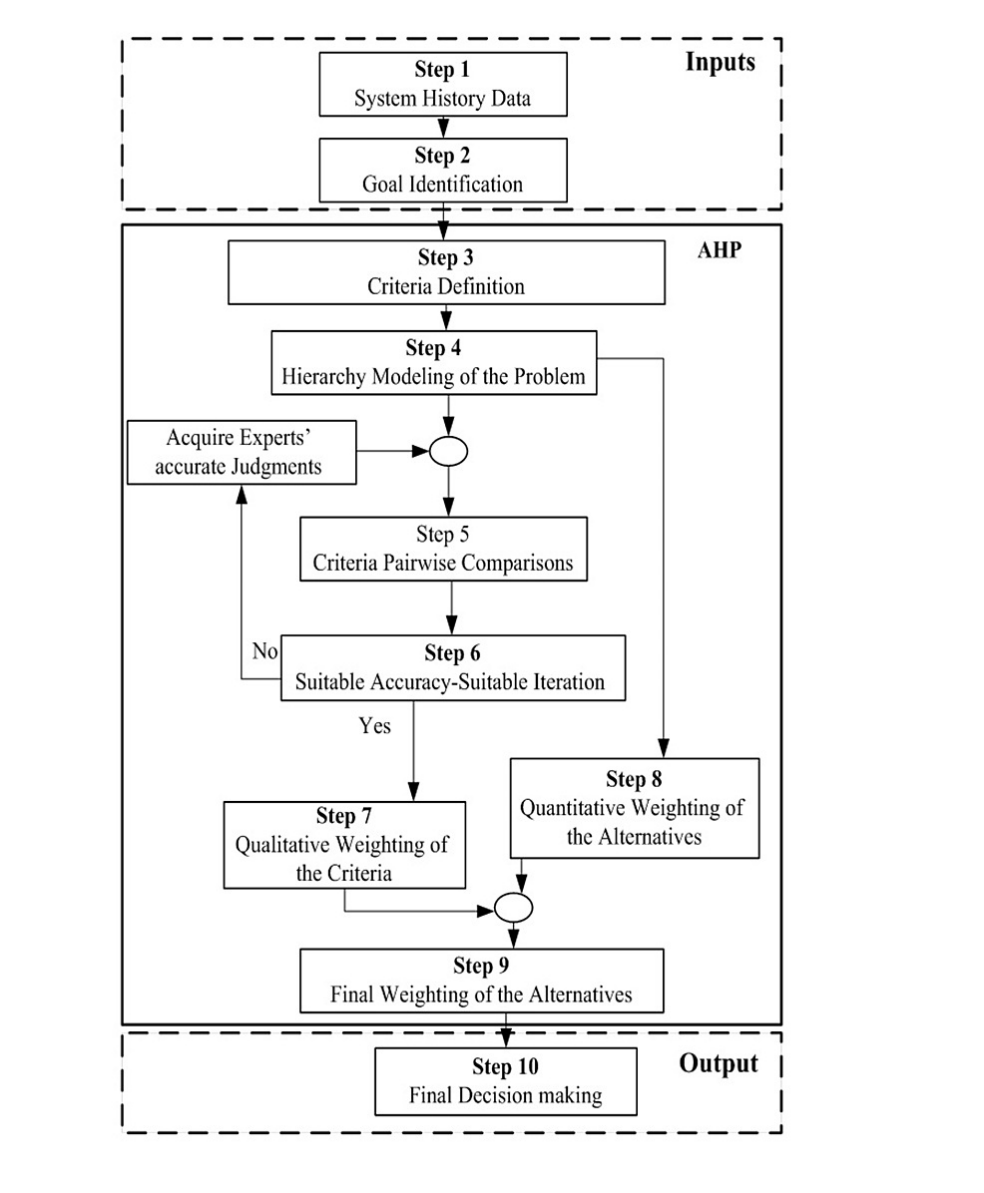
Decision-making process.

ANP consists of three phases, namely, creating a hierarchy structure, analyzing priorities, and assuring system compatibility. In the first step, the person who makes the decision would answer the comparisons based on his or her knowledge and experience. Incompatibilities may occur on some levels after intellectual comparisons are answered. Assuring compatibility is the next phase that assures compatibility between judgments by calculating the consistency rate. If the inconsistency rate is more than the allowed margin (0.1), the decision maker should rethink his judgments. After assuring compatibility between judgments, they can be combined and priorities of alternatives and criteria can be calculated. As shown in Figure 2, the first step includes system history data, which deals with collecting the essential technical and economic information associated with the network components. The second step deals with the desired goal accommodation that primarily leads the next steps.

The third step of ANP is to develop some effective criteria. Correspondingly, in the fourth, a hierarchy of the problem must be framed. The pair-wise comparisons of the determined criteria are performed subsequently (step 5). The collection of information is done in the form of the Delphi method, The Delphi method is repeated at least two times. The questionnaire was filled out by all experts, following which a preliminary analysis was done on the obtained data, and the results were achieved. Then, the questionnaire with the obtained results in the previous step were given to the experts, and each expert modified their previous opinion in case of need with regard to outcome results. This process, which is known as the Delphi method, continues until the results of two consecutive iterations become equal or reach an adequate precision (step 6). In steps 7 and 8, the weight of quantitative and qualitative criteria are gained. In step 9 the comparison between the alternatives for each criterion is discussed, and the results are obtained in step 10. The decision process stops here, and an appropriate ranking is specified for each dental irrigation. This study was conducted in two sections. The first was an in vitro evaluation of the antimicrobial properties of 100 mg/mL aqueous and ethanolic ginger extracts in comparison with NaOCl. The second part included the MCDM method.

Implementation of the ANP method

In this section, the implementation of the proposed method (ANP) has been investigated. After determining the goal, which is determining the priority ranking for mentioned endodontic irrigants, the appropriate criteria to address the best material were divided into four main categories (Table 3).

**Table 3:** Proposed criteria for prioritizing available locations.

Criteria	CRI. i
Antibacterial strength	CRI.1
Low risk	CRI.2
Cost-effective	CRI.3
Availability	CRI.4

Some criteria were divided into several sub-criteria. For antibacterial strength criteria, results for inhibition zone against *E. faecalis* and surface tension were considered as sub-criteria. The evaluation of alternatives was performed for each sub-criterion. Difficulty in accessing the ginger extract, NaOCl, and CHX was considered as a sub-criteria for the availability criteria. Based on a one-on-one comparison by 30 dentists, the value of these sub-criteria was achieved.

Reviewing the antibacterial criteria

Preparation of Ginger Extracts

In this study, 400 g of fresh ginger was procured from the local market of Shiraz, Iran, and was cleaned using distilled water. The cleaned ginger was then minced into fine pieces and soaked with 1,600 mL of distilled water (98% ethanol for ethanolic extract) for 24 hours at room temperature. The resulting extract was filtered through filter paper (Whatman number 1). During the filtration process, the residue of ginger and the filtrate were obtained. The filtrate was placed over a steam bath apparatus for a week to facilitate the evaporation of solution content from the filtrate. After a week, the dried extract was collected. The extraction efficiency of aqueous and ethanolic extract per 400 g of ginger powder was calculated as 10% and 5%, respectively (total yield was 40 g and 20 g for aqueous and ethanolic extract, respectively).

Preparation of 10% Stock Solution of Ginger Extract

In this study, 10 g of ginger extract powder was dissolved in 100 mL of distilled water (the inert solvent for ethanolic extract was 80% dimethyl sulphoxide (DMSO)) to obtain 10% ginger extract.

Microbiological tests

Standard culture of *E. faecalis* (ATCC 29212) was grown on Mueller Hinton agar (MHA) and incubated for 24 hours at 37°C as a test for sterility. A 10% stock solution of ginger extract, CHX (2%), NaOCl (5.25%, 2.5%, 1.125%), DMSO (80%), and distilled water were also used in the study. For this purpose, wells of 6.5 mm depth and 6 mm diameter were prepared, which were filled with 100 μL of each solution. Results were expressed as the mean and standard deviation for each solution. The analysis of variance was used to compare the diameter of the inhibition zone. P-values less than 0.05 (p < 0.05) were considered significant. Table 4 shows the zone of inhibition of 10% ethanolic and aqueous ginger extract against *E. faecalis*. Ethanolic ginger showed comparable results with 2.5% NaOCl and was significantly far from the strength of 5.25% NaOCl.

**Table 4:** Antimicrobial activity of ginger extract using the well method. SD = standard deviation; NaOCl = sodium hypochlorite; CHX = chlorhexidine; DMSO = dimethyl sulfoxide

		Wheel method (mm)
		Mean (SD)
	NaOCl 1.125 %	15.3 (0.70)
	NaOCl 2.5%	22.2 (0.45)
	NaOCl 5.25%	30.7 (0.78)
Control+	CHX 2%	33.75 (0.80)
Control-	Distilled water	-
Control-	80% DMSO	-
	10% aqueous ginger	14.8 (0.85)
	10% ethanolic ginger	19.95 (0.70)

Reviewing the risk criteria

Complications of NaOCl use, such as damage to clothing and surrounding tissues, allergic reactions, chemical burns and tissue necrosis, neurological damage, and upper airway obstruction, may occur with accidental spillage or extrusion beyond the root apex [20]. Most complications appear to be the result of its accidental injection beyond the root apex, which can cause immediate severe pain and swelling of the surrounding soft tissue, profuse bleeding from the root canal, hemorrhage of the skin and mucosa, secondary infection, and paresthesia [21]. Increasing the irrigation time with a lower concentration (1.0-2.5%) of NaOCl may be more suitable for endodontic irrigation to obtain an optimal antimicrobial effect with minimal risk of tissue-irritating injury. CHX is an alternative irrigant to NaOCl because of its broad-spectrum antimicrobial activity and lower toxicity than 5.25% NaOCl solution [22,23]. Several in vivo studies have proven the healing and anti-inflammatory effects of the ethanolic ginger extract [24]. Moreover, the solvent for ethanolic extract DMSO has a great ability to penetrate the skin and other biological membranes. DMSO has been used as a transportation device to pass small molecules through the skin since 1960. It was approved by the FDA for use in digestive drugs [25,26].

Reviewing the surface tension

**Figure 3:**
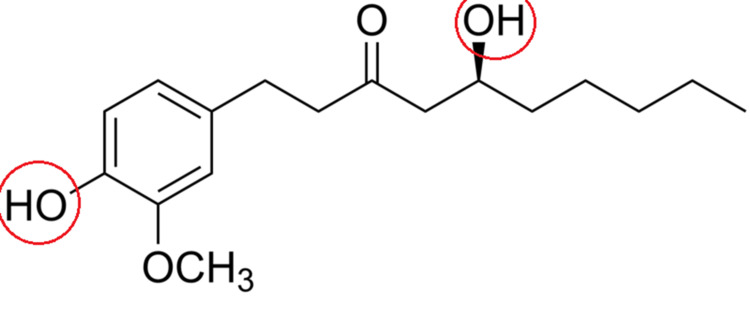
Structure of gingerol, an active component of ginger extract.

Because of the complexity of root canal morphology, some intra-radicular areas remain inaccessible to chemo-mechanical preparation. It has been further suggested that modification of the surface tension of irrigating solutions may improve irrigation efficacy by allowing irrigants to flow into remote areas as surface tension inhibits the spread of a liquid over a surface and its ability to permeate capillary tubes. A lower surface tension enhances the penetration into inaccessible areas of root canals and improves the overall effect of the solution. CHX (2%) showed the best wettability in relation to the other tested irrigant, followed by 2.5% NaOCl [27]. The surface tension of distilled water, which was used as a control, was measured to be 73 mN/m^2^. When two different concentrations of NaOCl solution were compared, it was observed that 2.5% NaOCl had lower surface tension than 5.25% NaOCl. The surface tension of 2% CHX was lower than 5.25% NaOCI [28]. The surface tension of 80% DMSO was 43 mN/m^2^. The addition of the 10% ginger extract also enhanced the hydrophilicity on the surface by lowering the water contact angle from 93° to 85°. The presence of oxygen-containing functional groups, such as hydroxyl (−OH) and carboxyl (−COOH), is well-known to improve the membrane hydrophilicity [29] because it creates strong intramolecular dipole moments. Therefore, hydroxyl (−OH) groups (Figure 3) in the ginger extract-based chemicals were expected to enhance the membrane hydrophilicity.

 Table 5 presents the surface tension for each solution.

**Table 5:** surface tension for each solution NaOCl = sodium hypochlorite; CHX = chlorhexidine; DMSO = dimethyl sulfoxide

Irrigant	Concentration	Surface tension (temperature 37°C)
Distilled water	-	70.56
Aqueous ginger extract	10%	>56
NaOCl solution	5.25%	56
	2.5%	45
	1.125%	56
CHX	2%	43
DMSO	80%	43
Ethanolic ginger extract	10%	<43

Reviewing the cost criteria

Table 6 shows the price per gallon for each solution (from a California local market), A pure ginger extract with high quality is not accessible worldwide. Due to the complexity of the technique and the material used, the costs involved in preparing ginger extract were significantly higher than that of the NaOCl group. Therefore endodontic treatment with NaOCl has a lower cost than ginger extract.

**Table 6:** Price per gallon for each solution (from a California local market).

Solution type	Price per gallon (4 L)
Chlorhexidine 2%	$21.99
Bleach, 5.25% sodium hypochlorite	$21.12
Bleach, 2.5% sodium hypochlorite	$17.01
Bleach, 1.125% sodium hypochlorite	$14.7
Ginger extract 10%	lower price = $40, upper price = $65

Reviewing the availability criteria

The global NaOCl market size was USD 261.7 million in 2020 and is projected to reach USD 385.6 million by 2028. The global chlorhexidine gluconate solution market size was estimated at USD 173.49 million in 2021 and is projected to reach USD 209.44 million by 2028 [30]. Ginger powder is easy to access but there is limited availability of fine ginger extract, which makes it difficult to find pure ginger extract in local markets.

Irrigation alternatives

The available alternatives are listed in Table 7.

**Table 7:** Alternatives for irrigation. NaOCl = sodium hypochlorite; CHX = chlorhexidine

Alternative	Solution
Aqueous ginger extract	Sol. 1
Ethanolic ginger extract	Sol. 2
NaOCl 1.125%	Sol. 3
NaOCl 2.5%	Sol. 4
NaOCl 5.25%	Sol. 5
CHX 2%	Sol. 6

## Results

The evaluation of alternatives in each criterion

The pair-wise comparisons of the determined alternatives for any criteria and the weight of each criterion define the priority of materials. After combining the experts’ answers and the sub-criteria together, the final weights and priorities in each criterion were determined, as shown in Table 8. With a pair-wise comparison between the alternatives based on the experts’ opinions in qualitative criteria and considering the available documents in quantitative criteria according to the provided algorithm in Figure 2, the final weight of the alternatives is specified in Table 8.

**Table 8:** Final weights for any possible location based on each criterion. Sol. 1 = aqueous extract; Sol. 2 = ethanolic extract; Sol. 3 = 1.125% NaOCl, Sol. 4 = 2.5% NaOCl; Sol. 5 = 5.25% NaOCl; Sol. 6 = 2% CHX. NaOCl = sodium hypochlorite; CHX = chlorhexidine

CRI. i	CRI.1	CRI.2	CRI.3	CRI.4	Final weight	Ranking
Weight of criteria	0.704	0.117	0.099	0.080	---	---
Sol. 1	0.056	0.288	0.072	0.061	0.096	6
Sol. 2	0.140	0.288	0.053	0.055	0.143	4
Sol. 3	0.069	0.170	0.350	0.293	0.121	5
Sol. 4	0.145	0.096	0.245	0.244	0.157	3
Sol. 5	0.277	0.060	0.145	0.195	0.234	2
Sol. 6	0.307	0.098	0.133	0.152	0.248	1

The ranking is determined based on the priority percentage for each irrigant. As can be seen, 2% CHX (Sol. 6) with the highest preference is defined as the output of the analytical analysis. The criteria, which cause the success of CHX in competition with other alternatives, are antibacterial strength and availability. With respect to the high weighting of these criteria, despite having a lower weight in risk factors and cost, CHX is selected as the best alternative. Due to great wettability and being risk-free, ethanolic ginger extract got a final weight close to 2.5% NaOCl, while the aqueous extract showed disappointing results next to 1.125% NaOCl.

The analysis was performed with great precision. The inconsistency rate, expressed as the CR index, was calculated to be very low at 0.004.

## Discussion

**Figure 4:**
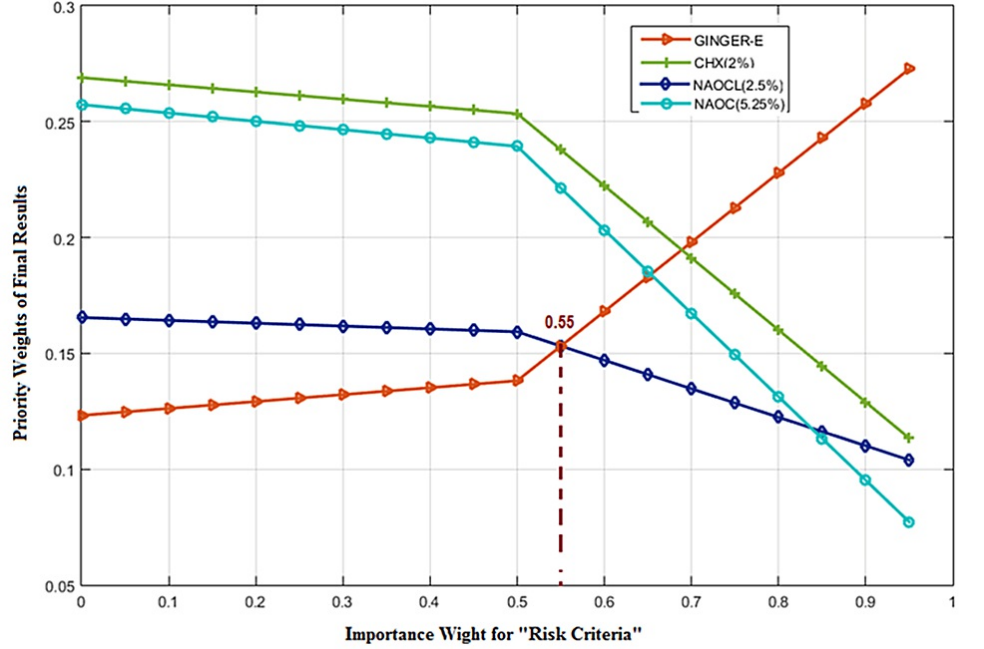
Sensitivity analysis to assess the effects of weight change in the risk criteria. GINGER-E = ethanolic ginger extract; NaOCl = sodium hypochlorite; CHX = chlorhexidine

Bacteria and their byproducts play an essential role in the initiation and progression of peri-radicular diseases and may be responsible for causing failures after root canal treatment [31]. Their persistent presence in apparently well-treated root canals may disrupt the restorative processes after treatment. Numerous bacteria have been isolated from dental canal infections, with *E. faecalis* being one of the resistant bacteria. Moreover, several studies have shown that this bacteria is one of the most common species isolated after root canal treatment [32]. This species is the most common bacteria identified from treated root canals that cause chronic apical periodontitis. The ability of this bacterium to invade the dentinal tubules, resistance to various environmental conditions of the canal, and adaptation to the unfavorable conditions inside the canal are among the factors that make this bacterium a pathogenic infection resistant to endodontic treatments [33]. The therapeutic potential of plant extracts as detergents and drugs inside the root canal has received special attention in recent years [34]. Ginger has acceptable antimicrobial effects, with active compounds such as gingerol and shogaol. These are lipid-soluble phenol compounds primarily isolated from the root of ginger. Its bactericidal and bacteriostatic properties can break the phospholipid membrane to become permeable due to the released main components of the cell and inhibit membrane function [35]. On an evaluation of different irrigating solutions for smear layer removal, 5.25% NaOCl was more efficient than ginger extract [36]. An in-vitro study [37] indicated that 2% CHX showed high antibacterial activity against *E. faecalis*, followed by calcium hydroxide and ginger extract. In this study, 10% ethanolic ginger extract showed antimicrobial activity against *E. faecalis*, exhibiting a mean zone of inhibition of 19.9 mm at 100 µL according to the well method. 10% ethanolic ginger compared to 2.5% NaOCl, which showed a zone of inhibition of 22 mm, indicated that these two are analogous. To come to a decisive conclusion between these endodontic irrigants, wide standards and criteria need to be taken into account. In this study, a method for the optimum ranking of root canal solutions has been presented. The proposed method is ANP, which is an MCDM subset. After determining the appropriate criteria, it is important to apply the opinions and experiences of experts in the endodontic field. There are several techniques for gathering and integrating experts’ opinions; the Delphi technique was used in this study [38]. The proposed method (ANP) for several alternatives was investigated; 5.25% NaOCl and 2% CHX were at the top of the list. There is a lack of robustness in the findings or conclusions based on primary analyses of data in any experimental study. Conducting sensitivity analysis provides numerous benefits for decision-makers. First, it acts as an in-depth study of all variables. Because it is more in-depth, the predictions may be far more reliable. Second, it allows decision-makers to identify where they can make improvements in the future. It can help gain insight into which assumptions and parameters are critical. The process involves changing some input variables and traces the output behavior. It may be thought that the larger the weight of a criterion, the more criticality is assigned to that criterion. However, this may not be the case. The criterion would be considered critical when a small change in its weight would cause a significant change in the final result. Hence, it is possible for the criterion with a rather small weight of importance to be more critical. It can be observed in Figure 4 that the variation in the weight of the risk criteria (low-risk) from zero to 1 per unit (PU) will change the recommended irrigation for root canal therapy.

**Figure 5:**
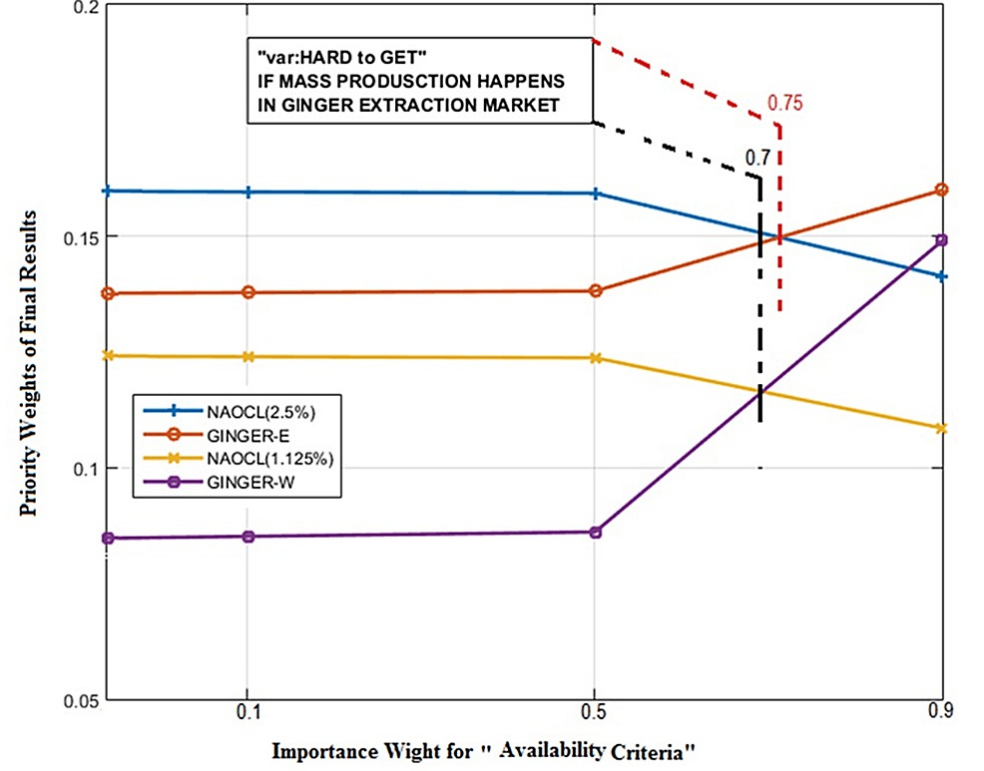
Sensitivity analysis based on accessibility of each solution. GINGER-E = ethanolic ginger extract; GINGER-W: water-based (aqueous) ginger extract; NaOCl = sodium hypochlorite

The vertical line in Figure 4 represents the main weight of risk criteria. 2.5% NaOCl has been ranked above the ethanolic ginger extraction, but when the weight of criteria goes beyond 0.55 PU, ethanolic ginger extraction is the better alternative. The same can be seen in Figure 5. If mass production and more accessibility of ginger extraction occur, it can surpass 2.5% NaOCl for the importance weight higher than 0.75 in availability criteria.

**Figure 6:**
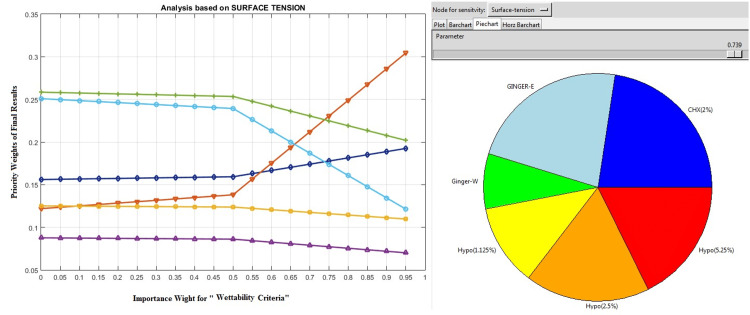
Sensitivity analysis based on surface tension. GINGER-E = ethanolic ginger extract, GINGER-W = water-based (aqueous) ginger extract; NaOCl = sodium hypochlorite

The surface tension was a sub-criteria of antibacterial strength. Given experts’ consideration, the sensitivity analysis for this criteria showed that (Figure 6) with an importance of .0739 and above, the 10% ethanolic ginger extract can overcome other endodontic solutions due to its superior level of wettability.

## Conclusions

To carefully prioritize endodontic irrigants, a wide range of standards and criteria should be considered. In this paper, a method for the optimum ranking of several dental solutions is presented. The proposed method is ANP. After determining the appropriate criteria and including the opinions and experiences of experts in the ANP framework, results indicate that with respect to the low risk and great wettability of the ethanolic ginger extract, it provides comparable and analogous results to 2.5% NaOCl.
